# Soft Computing of a Medically Important Arthropod Vector with Autoregressive Recurrent and Focused Time Delay Artificial Neural Networks

**DOI:** 10.3390/insects12060503

**Published:** 2021-05-31

**Authors:** Petros Damos, José Tuells, Pablo Caballero

**Affiliations:** 1 Department of Community Nursing, Preventive Medicine and Public Health and History of Science, Faculty of Health Science, University of Alicante, 03080 Alicante, Spain; 2 University General Hospital of Thessaloniki, AHEPA, Kiriakidi 1, 54621 Thessaloniki, Greece; tuells@ua.es (J.T.); pablo.caballero@ua.es (P.C.)

**Keywords:** mosquito population system, *Culex* sp., NARX model, FTD model, decision making, public health

## Abstract

**Simple Summary:**

Arthropod vectors are responsible for transmitting a large number of diseases, and for most, there are still not available effective vaccines. Vector disease control is mostly achieved by a sustained prediction of vector populations to maintain support for surveillance and control activities. Mathematical models may assist in predicting arthropod population dynamics. However, arthropod dynamics, and mosquitoes particularly, due their complex life cycle, often exhibit an abrupt and non-linear occurrence. Therefore, there is a growing interest in describing mosquito population dynamics using new methodologies. In this work, we made an effort to gain insights into the non-linear population dynamics of *Culex sp*. adults, aiming to introduce straightforward soft-computing techniques based on artificial neural networks (ANNs). We propose two kind of models, one autoregressive, handling temperature as an exogenous driver and population as an endogenous one, and a second based only on the exogenous factor. To the best of our knowledge, this is the first study using recurrent neural networks and the most influential environmental variable for prediction of the WNv vector *Culex sp.* population dynamics, providing a new framework to be used in arthropod decision-support systems.

**Abstract:**

A central issue of public health strategies is the availability of decision tools to be used in the preventive management of the transmission cycle of vector-borne diseases. In this work, we present, for the first time, a soft system computing modeling approach using two dynamic artificial neural network (ANNs) models to describe and predict the non-linear incidence and time evolution of a medically important mosquito species, *Culex* sp., in Northern Greece. The first model is an exogenous non-linear autoregressive recurrent neural network (NARX), which is designed to take as inputs the temperature as an exogenous variable and mosquito abundance as endogenous variable. The second model is a focused time-delay neural network (FTD), which takes into account only the temperature variable as input to provide forecasts of the mosquito abundance as the target variable. Both models behaved well considering the non-linear nature of the adult mosquito abundance data. Although, the NARX model predicted slightly better (R = 0.623) compared to the FTD model (R = 0.534), the advantage of the FTD over the NARX neural network model is that it can be applied in the case where past values of the population system, here mosquito abundance, are not available for their forecasting.

## 1. Introduction

The mosquito species is considered as one of the most important arthropods of several vector-borne diseases (VBDs) caused in humans, companion animals, and livestock [1]. Mosquito species are present in more than half of the world’s population’s living areas, and therefore, to prevent outbreaks of related disease, sustained mosquito control efforts are important [1]. However, although a wide variety of arthropod-borne diseases are transmitted by mosquitos [2], only a limited number of species play a primary role in vector-borne epidemiology and in the outbreak of neglected tropical diseases, such as the West Nile Virus (WNV). In particular, mosquitoes of the genus *Culex* are generally considered as the principal vectors of WNV [3]. The WNV is maintained in mosquito populations through vertical transmission (adults to eggs) and further transmitted through the life cycle between mosquito and animal hosts, with the predominant reservoir being birds [4,5].

In southern Europe, the WNV has been detected in the indigenous mosquito species, *Culex pipiens*, including Italy and Portugal [3,6,7]. Moreover, in Greece, *C. pipiens* has been identified as the dominant and endophilic species in rural areas in central Macedonia, including the prefectures of Imathia, Kilkis, Pella, Pieria, and Thessaloniki, while having been identified as the major vector of the WNV outbreaks [8]. To date, since the beginning of 2019, EU member states have reported 463 human WNV infections, including in Greece (223, 34 deaths among them), Romania (66), Italy (53), Hungary (36), Cyprus (16), Bulgaria (5), Austria (4), Germany (4), France (2), and Slovakia (1) [9]. Much effort and money has subsequently been exerted for the health care of patients infected with vector-borne diseases and to mitigate the effects of vector-borne diseases [10].

Nevertheless, the principal method by which VBDs, and WNV in particular, are managed is through vector control. Actually, mosquito vector control has been responsible for a greater suppression in the distribution of VBDs than drugs and vaccines [11]. To date, the World Health Organization (WHO), in its recent 2017–2030 Vector Control Response (GVCR) strategy, called for effective, locally adapted and sustainable vector control [12]. Therefore, a preventive and rational strategy for dealing with vector-borne diseases should be based on the elimination of the vector agents rather than the disease itself [11,12]. Aided by this is also the fact that the association between mosquito abundances and human disease instances is delayed.

However, due to the climate-related abrupt dynamics and complex nature of the mosquito life cycle, there is an absence of field-based decision tools for understanding local vector behavioral ecology and to be used for tailored mosquito control [13,14]. Furthermore, understanding the temporal evolution of mosquito vectors and related disease incidences is critical for targeting limited prevention, surveillance, and control resources (e.g., temporal targeting of vaccination, drug administration, or education campaigns; use of sentinel sites to monitor vector abundance; and identifying critical time periods for most effective use of pesticides).

Most often, to predict mosquito abundances, there have been dynamic (or mechanistic) models developed [15,16]. However, such deterministic models require complicated domain knowledge, and the reliability depends strongly on the availability of parameters which, most often, are not known for each of the mosquito growth stages and life transitions. For instance, the mosquito life cycle is rather complex, since immature stages are waterborne and difficult to observe compared to adults [17,18,19]. On the other hand, the use of traditional regression models, which are mostly used to associate climate variables to vector abundances, although useful in detecting significant correlations, do not provide any dynamic association and, therefore, cannot be used as prognostic tools of mosquito abundance *per se*.

One other disadvantage of the statistical regression model, as previous studies have shown when applied on noisy mosquito data [20], is that it is limited by its use of linear relations and normality assumptions to estimate its parameters. Hence, due to the stochastic nature of the mosquito population, their dynamics have also been studied through time series analysis [21,22,23]. However, due to impacts of various internal and external factors, such processes are of a nonlinear nature, and therefore, it is generally difficult to analyze and predict population dynamics using linear autoregressive models [24].

Recent trends have proven that soft computing techniques, like artificial neural networks (ANNs), are becoming popular as alternatives for conventional time series analyses and data modeling in the health care domain [25,26]. Particularly, soft computing modeling approaches have the ability to adapt themselves according to the problem domain, thus providing a good balance between exploration and exploitation processes [26,27]. ANNs are flexible nonlinear systems that show robust performance in dealing with noisy or incomplete data [28]. Additionally, they have been proven very utile when the relationships between the variables are complex, multidimensional, and nonlinear, as found in complex biological systems [29]. Therefore, ANNs have the ability to generalize from the input data and may be better suited than other modeling approaches to predict the non-linear dynamics of mosquito abundance outcomes.

To date, ANNs have been applied in many fields of science, *inter alia*, including medicine [30,31], economics [32], chemistry [33], environmental modeling [34,35], ecology [36,37], mosquito species identification [38], and, very recently, in pest management [39], as well as predicting malaria abundances [40]. Moreover, ANN are increasingly being used to inform health care management decisions [41,42]. The advancement of computer technologies has permitted the development of powerful tools, which provide a standardized way to collect data, opening new perspectives for expert systems’ development and decision making [43].

However, there are very few cases in which they have been applied to model arthropod population dynamics [44] and even fewer in modeling mosquito dynamics [40,45]. Chon et al. (2000) [44] have applied these models to forecast dynamic data of a pine tree forest pest population. In this approach, the backpropagation algorithm was implemented on multilayered data, in which changes in population density were sequentially given as input, whereas densities of the subsequent samplings were provided as matching target data for training of the network. Nevertheless, a limitation of the standard backpropagation algorithm is that it creates spatial analogues of temporal patterns, which have a static rather than a dynamic nature [46,47].

The purpose of this article was to develop, implement, train, and validate ANNs to describe, for the first time, the temporal evolution of mosquito vectors and capture the non-linear dynamics of the *Culex* spp. adult population, especially. Based on previous studies, which have explored the effect of exogenous (climate) as well as endogenous (populations) variables in the dynamics of *Culex* sp. [20,48,49], we now developed two ANNs models to describe and predict its population dynamics.

The study hypothesis was based on the fact that adult mosquito abrupt dynamics depend significantly on the initial structure of mosquito dynamics (i.e., previous population values), as well as that temperature needs to be taken into account when modeling mosquito population dynamics and planning public health policies. Particularly, we applies, for the first time, two non-linear autoregressive ANNs in modeling the population dynamics of *Culex* sp., a major vector of WNV. The first one was an exogenous non-linear autoregressive neural network (NARX), which was designed to take as inputs the temperature as an exogenous variable and mosquito abundance as an endogenous variable, whilst the second was a focused time-delay neural network (FTD), which took into account only the temperature variable as input to provide forecasts of the mosquito abundance used as the target variable.

We consider this study important since it not only provides information on the functioning of population dynamics, but also presents a framework that is utile for the development of expert systems. With the mosquito abundance prediction, particularly, public health authorities could predict the time evolution of mosquito abundance which is a prerequisite for successful management.

## 2. Materials and Methods

### 2.1. Mosquito Surveillance and Temperature Data

To develop, validate, and test the NARX and the FTD neural network models, we used free mosquito trap data available from the open European Union Data Portal (EU ODP) (http://data.europa.eu, accessed on 3 May 2019) [50], which provides access to an expanding range of data from the European Union (EU) institutions and other EU bodies, which can be reused for commercial or non-commercial purposes (European Commission Decision, 2011/833/EU). In particular, details on the available mosquito trap data were linked to former studies that investigated the associations between climatic factors and the West Nile Virus-infected mosquitoes during the first period of the WNV outbreak in Greece that occurred in 2011 and, mostly, in 2012 [51]. We used adult mosquito trap data of *Culex* spp. sampled from 11 closely related locations in central Macedonia and Greece, which have the same habitat characteristics. Data were handled as vectors, which consisted of close-to-weekly time intervals of the number of adult mosquitoes captured in CO2 traps from mid-May until September and during two successive observation years (2011 and 2012). 

Because of slight differentiations between the times intervals of some of the trap counts, data were transformed to mosquitoes per trap per day (MTD) and, thereby, were averaged over the 11 nearby sampling locations [20]. The MTD thus estimates the average number of mosquitoes captured on the day that the trap was exposed in the field.

Climate data, and in particular, mean air temperatures, were obtained by the national observatory of Athens through a meteorological station, which was located in Makrohori town, which was in the same level and nearby the mosquito observation area (http://stratus.meteo.noa.gr/front, accessed on 2 April 2020) [52].

### 2.2. Formulation of the NARX and the FTD Neural Networks

We first applied a standard autoregressive network with exogenous inputs (NARX), which was part of discrete-time non-linear systems, which conceptually had feedback connections, which enclosed the layers of the network and used the past values for prediction [53]. The FTD neural network may be considered as a simplified version of the NARX in which the output feedback is eliminated (see below). The defining equation for the NARX model with a parallel architecture can be expressed as follows [54,55]:

(1) $$y\left(t\right)=F(\left[y\left(t-1\right),y\left(t-2\right),\ldots ,y\left(t-d_{y}\right),u\left(t-1\right),u\left(t-2\right),\ldots ,u\left(t-d_{u}\right)\right]$$

where F(·) is the mapping (unknown non-linear) function of the neural network, *y*(*t*) is the output of the NARX at time step t, $y\left(t-1\right),y\left(t-2\right),\ldots ,y\left(t-d_{y}\right)$ are the past output values of the NARX, $u\left(t-1\right),u\left(t-2\right),\ldots ,u\left(t-d_{u}\right)$ are the exogenous inputs of the NARX, $d_{u}$ is the number of input delays, and $d_{y}$ is the number of output delays. Thus, the output of the NARX network y(t) is fed back (closed loop) to the input of the network through delays *t*, and thus, Equation (1) can be described in a compact form as follows [56]:

(2) y(t)=F([y(t),u(t)]),

where y(t)∈ℝ and u(t)∈ℝ denote the output (mosquito abundance) and the input (temperature) of the model at described time t, respectively, for different lagged output and input memory orders. Moreover, a NARX neural network is usually trained in series-parallel (SP) mode first and, later on, in a parallel (P) mode. To date, in the SP mode, only the actual values are taken into account and form the outputs as follows:

(3) $$\begin{array}{cc} \hat{y}\left(t\right)=\hat{F}([y\left(t-1\right), & y\left(t-2\right),\ldots ,y\left(t-d_{y}\right),u\left(t-1\right),u\left(t-2\right),\ldots ,u\left(t-d_{u}\right)]\\ & =\hat{F}\left(\left[y_{SP}\left(t\right),u\left(t\right)\right]\right) \end{array}$$

In the P mode, the outputs that are estimated are fed back to the network and are included in the outputs:

(4) $$\hat{y}\left(t\right)=\hat{F}(\left[\hat{y}\left(t-1\right),\hat{y}\left(t-2\right),\ldots ,\hat{y}\left(t-d_{y}\right),u\left(t-1\right),u\left(t-2\right),\ldots ,u\left(t-d_{u}\right)\right]=\hat{F}\left(\left[y_{P}\left(t\right),u\left(t\right)\right]\right)$$

In the case of the FTD neural network, the output memory of a NARX model is set by a zero delay (*n_y_* = 0), resulting in a plain neural network architecture which can be described as follows:

(5) $$y\left(t\right)=F\left[u\left(t-1\right),u\left(t-2\right),\ldots ,u\left(t-d_{u}\right)\right]=F(\left[u\left(t\right)\right])$$

where u(t)∈ℝ is the input regressor (here, temperature). Thus, the FTD neural network is a simplified formulation of the NARX model that discards all the dynamic learning information of the output past memories.

### 2.3. Architecture and Components of the Neural Networks

Both, the NARX and the FTD neural networks consist of the input layer and the output layer, which approximate the map function F(·) through an internal architecture known as multi-layer perceptron (MLP). By definition, the classical MLP consists, at least, of three layers: the input, the hidden, and the output layer. If i is the number of neurons in the layer, and j is the number of elements in input vector $p_{j}$, then each vector of the input layer is connected to each neuron input trough the weight matrix W [57]:

(6) W=[w1,1 w1,2… w1,jw2,1 w2,2… w2,j  wi,1 wN,2… wi,j],

Since, in most cases, the number of inputs to a layer may differ from the number of neurons, the matrix is not necessarily nxn. For each single layer, each neuron multiplies the input layer $p_{i}$, given by the previous layer, by the weight vector $w_{i,j},$ which yields the following scalar product: $p_{j}\cdot w_{i,j}$ [57]. The weighted sum of the inputs (netsum) consists of the transfer potential, θ, which aggregates the inputs and its weights as follows:

(7) $$\theta =\overset{n}{\underset{\iota =1}{\sum }}p_{j}w_{i,j}\;$$

The transfer potential passes through a predefined activation function, f, to obtain the output, $a_{i},$ of the following neuron [53]:

(8) $$a_{i}=f\left(\overset{n}{\underset{\iota =1}{\sum }}p_{j}w_{i,j}+b\right),$$

where i is the index of the neuron in the layer, j is the input index of the neural network, and b is a bias vector. The output of the NARX neural network has a hyperbolic tangent sigmoid transfer (tansig) function in the inner layer and a pure linear function (purelin) in the output layer, which are given as follows:

(9) $$f\left(\theta \right)=\tan\mathrm{sig}\left(\theta \right)=\frac{2}{1+e^{-2\theta }}-1,$$

(10) f(θ)=purelin(θ)=θ

The equations which describe the function of the first and the second layers of a NARX and FTD neural network are as follows [58]:

(11) $$a1\left(t\right)=\overset{j}{\underset{i=1}{\sum }}w_{\mathrm{ij}}p1\left(t-d1\right)+b1,$$

(12) $$a2\left(t\right)=\overset{j}{\underset{i=1}{\sum }}w_{\mathrm{kj}}a1\left(t\right)+b2\;$$

where i and k are the number of neurons, $w_{\mathrm{ij}}$ is the weighted input of the network, p1(t−d1) are the lagged inputs of the layer 1, a1(t) is the output of the hidden node,$\;w_{\mathrm{kj}}$ are the weights of the second layer, and a2(t) is the output of the kth neuron in the lth layer at the time (t).

### 2.4. Model Training, Testing, and Validation

In the applied NARX model, the predictions of mosquito dynamics were performed from the past predicted values of the abundance time series and from the present and past values of the exogenous temperature input. To date, to extract these two key input variables we initially analyzed the correlation coefficients of different meteorological data with an imposed time lag. Moreover, we used 10 hidden neurons and 2 for the number of time-delays in weeks, because they gave satisfactory results after a preliminary training and testing of different combinations of hidden neurons and delays. Data division was performed randomly using both data sets (2011 and 2012), in which, finally, 60% of the data was used for NARX training (38 target time-series steps), 20% for validation (13 target time-series steps), and 20% for testing (13 target time-series steps). To date, the validation datasets consisted of the sample of data held back from training, while the test dataset was used for fine tuning (optimizing) the ANN model hyper-parameters (i.e., taking weights of the trained ANN and using it as initialization for a new model being trained, etc.). Each time step corresponded to the weekly counts of the *Culex* sp. mosquito abundances. The Levenberg–Marquardt (LM) algorithm was used as a training algorithm, in which the network training automatically stops when generalization stops improving, as indicated by an increase in the mean square error (mse) of the validation samples, which is used as cost function, C:

(13) $$\;C\left(w,b\right)=\frac{1}{n}\overset{n}{\underset{i=1}{\sum }}{\left(e_{i}\right)}^{2}=\frac{1}{n}\overset{n}{\underset{i=1}{\sum }}{\left(y_{i}-y_{j}\right)}^{2}\;$$

where w and b refer to all the weights and biases in the network, respectively, n is the number of training inputs, and $y_{j}$ are the outputs when $y_{i}$ is the input. The LM algorithm minimizes C as much as possible by optimizing weights and biases through gradient descent. The partial derivatives of the cost function with respect to any weight w and any bias b were estimated through a backpropagation algorithm.

All data analysis was performed using Matlab numerical computing environment and ANNs Simulink toolbox, and related programing language was developed by Mathworks [59].

## 3. Results

### 3.1. Network Architecture

**Figure 1:**
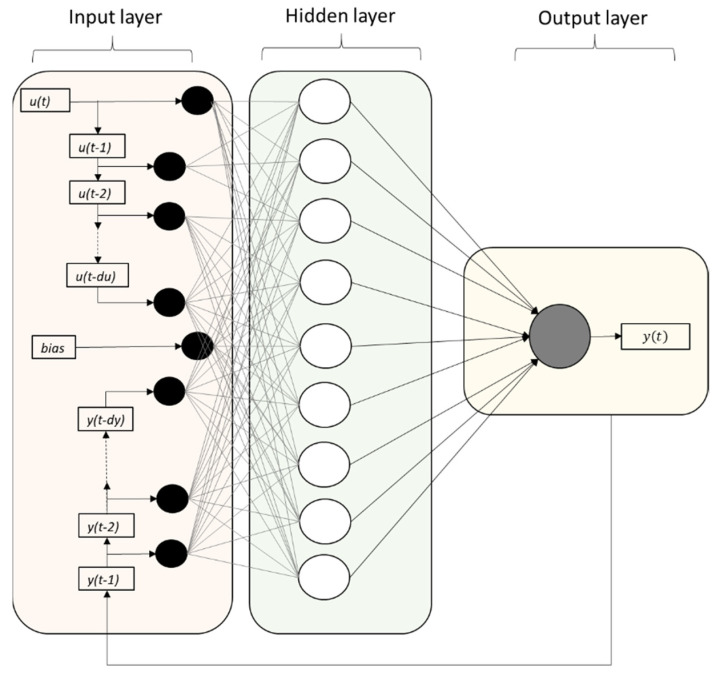
Graphical illustration of the NARX network with du input and *dy* output memory and a number of neurons in the hidden layer. Note that if the output memory is set at *dy* = 0, then the NARX network is reduced to a plain FTD neural network architecture.

The NARX neural network is a nonlinear auto-regressive model with exogenous inputs. Figure 1 is a graphical illustration of a NARX network, in a parallel identification mode, with du input and $d_{y}$ output delays. The NARX neural network structure has an input layer, which consists of the mosquito abundance counts and the temperature recording counts, which are connected through the weight matrix to each of the 10 neurons, which consist of the hidden layer. The model has been generated for two input delays of 1 and 2 weeks, respectively, for each of the two variables (mosquito counts and mean temperatures). The results of the hidden layer are linked through the summation function in the output layer.

**Figure 2:**
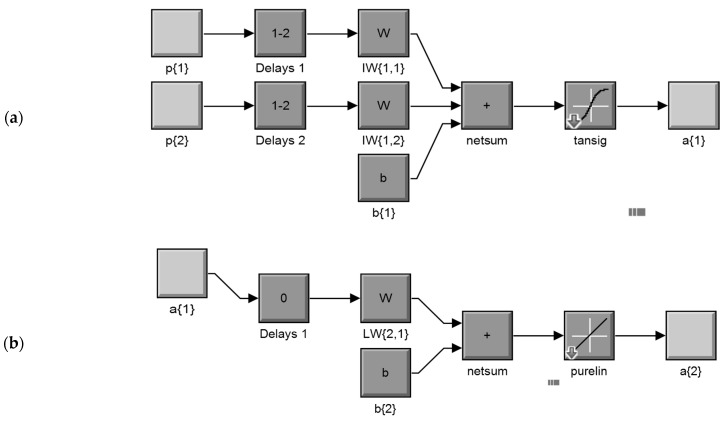
Abbreviated dynamic model structure, in a parallel mode, of the overall NARX network for the input layer (**a**) and the output layer (**b**), according to the Mat Lab Simulink ANN system model construction process (details in text).

An abbreviated dynamic model structure, in a parallel mode, of the overall NARX neural network for the input layer (a) and the output layer (b), according to the Mat Lab Simulink ANN system model construction process, is shown in Figure 2. In this structure, the network simulation data (the input of the model) consists of 2 concurrent vectors: *p*1 = {12} and *p*2 = {21}, where *p*1 is the mosquito abundance vector, which corresponds to weekly counts of *Culex* sp. adult stages, and *p*2 is the respective mean temperature vector. The FTD neural network architecture has the same topology as the NARX model but without the lagged mosquito input variable, and therefore, it consists of a feedforward network with a tapped delay line at the input.

The model was applied to predict the population (a{2}) of a medically important mosquito species (*Culex* sp.), from previous temperature recording values (delays 1) of exogenous inputs (p{1}) and previous (delays 2) mosquito population values (p{2}). Each element of the input and output network was connected to each neuron through a weighted matrix (W). Elements of layer 1, such as its bias (b{1}), net input, and output have a superscript 1 to indicate that they are associated with the first layer, while those of layer 2 have superscript 2. The FTD neural network has the same topology without the p{2} mosquito input variable, as well the related delays and weights (netsum: transfer potential θ, tansig: hyperbolic tangent sigmoid transfer function, purelin: pure linear function transfer function).

### 3.2. Model Training and Validation

**Figure 3:**
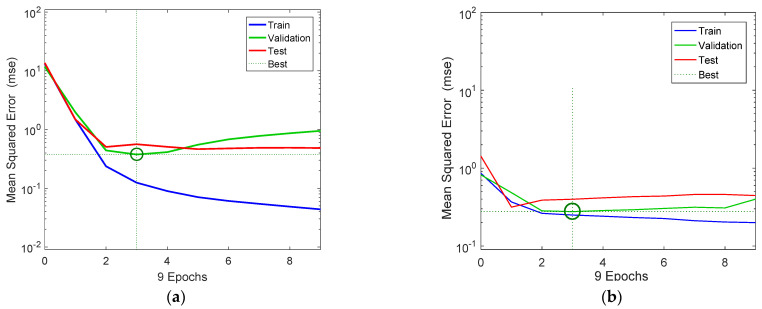
NARX (**a**) and FTD (**b**) neural network training, validation, and testing performance. Note that the best validation performance for the NARX model was 0.388 at epoch 3, and for the FDR model, it was 0.276 at epoch 3.

Figure 3a,b show the variation of the mse of the training, validation, and test data in respect to the successive number of iterations (epochs) for the NARX and the FDR neural network models, respectively. The three curves had a similar overall trend, except for the train data. Moreover, it can be seen that training and validation errors for the NARX model decreased until the highlighted epoch, and the best validation performance state was at 0.388 at epoch 3, in which the mse was minimized. Additionally, considering that validation error did not increase before this epoch, this indicates that overfitting has not occurred. The mse of the test data had a similar pattern, and it was minimized after 4 iterations and remained stationary after that point, which indicates that the model had reached its optimal state. However, the best states for the train data occurred after 3 time steps (epochs), at which the mse of the test data was gradually minimized.

**Figure 4:**
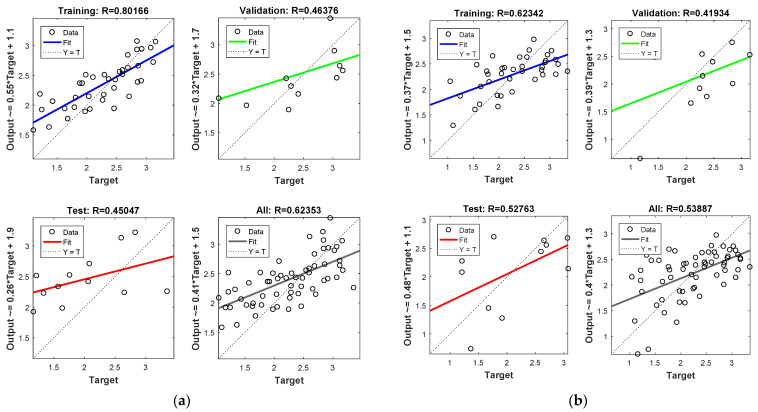
NARX (**a**) and FTD (**b**) neural network training, validation, and testing performance. Note that the best validation performance for the NARX model was 0.388 at epoch 3, and for the FDR model, it was 0.276 at epoch 3.

Figure 4 shows model performance in terms of regressions between the output and the target data sets (i.e., training, validation, testing, and overall) for the NARX (Figure 4a) and the FTD model (Figure 4b). In most cases, the model performed well considering that the data were in the in the vicinity of the diagonal. The correlation coefficient was at acceptable levels in both cases and in respect to the available data set (R = 0.623 and R = 0.534 for the NARX and FTD models, respectively). Moreover, considering the non-linear and abrupt nature of the mosquito data, the overall model predictions were in acceptable levels when comparted to the actual abundance data. In addition, it should be mentioned that the model performance was considerably higher by taking into account only the training data (i.e., r = 0.8 and r = 0.62, for the NARX and FDR models, respectively) and that the final overall model performance values were affected by the lower validation and model testing performances. Thus, we expect that the model performance could be considerably improved if the test dataset size was higher. However, to make the network model more efficient, we decided to keep a larger data set to be preprocessed for training, despite the smaller returns that were shown for the testing and validation performances.

### 3.3. Overall Model Performances

**Figure 5:**
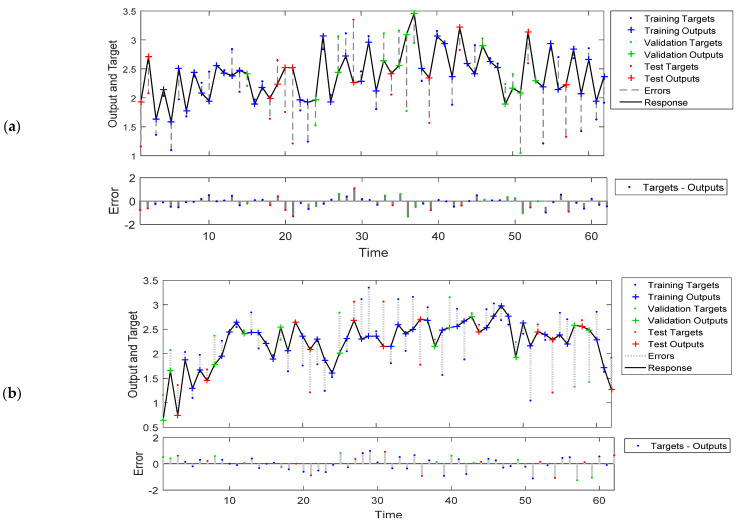
Response of the NARX (**a**) and FTD (**b**) neural network model output to the mosquito population time series and error. The model training was performed in an open loop (i.e., parallel series architecture), including the validation and testing step, and later on, after training, it was transformed to a closed loop for the multistep-ahead prediction.

Figure 5a depicts the response of the NARX neural network model output to the mosquito population time series (upper part), as well as the error of the output (lower scheme), while Figure 5b depicts the response of the FTD neural network model output to the mosquito population time series (upper part), as well as the error of the output (lower scheme). The time scale corresponds to weekly time intervals (from mid-May until September). In general, the prediction-output trend performed well, although there were time steps where the prediction results were not ideal, and the reason for that is that the amount of available data was relatively small. However, for the first model (NARX model), there were some cases which showed high values with low target values and thus positive bias, while in the second (FTD) model, there were few low output values with high target values, suggesting a negative bias. Nevertheless, in most cases, the deviations during certain time steps were in the range of −1.4 to 1.3, which is relatively low, and the distribution was around zero.

**Figure 6:**
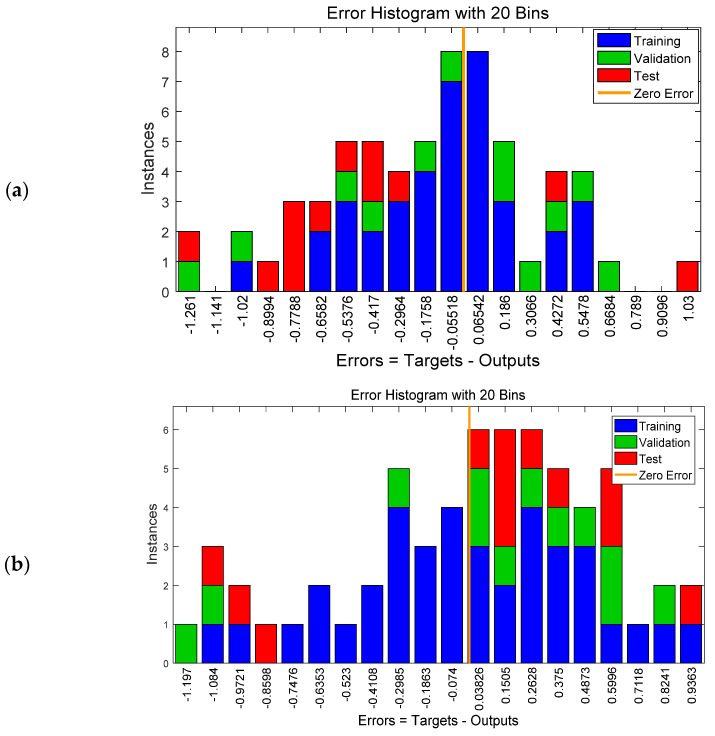
Error histogram chart having 20 bins for the NARX (**a**) and the FTD (**b**) neural network.

Moreover, the overall frequency of the error term is shown in Figure 6, which is an error histogram chart having 20 bins. The number of samples from each data set is represented by a vertical bar. The error of the NARX neural network ranged from −1.2 (leftmost bin) to 1.03 (rightmost bin), while the error of the FTD neural network ranged from −1.1 (leftmost bin) to 0.9 (rightmost bin). For both models, and especially for the NARX model, the vast majority of the training outputs had a smaller error and were slightly between−0.4 and 0.4. This is due to the fact that the set used for training contained more data (i.e., 60% of data) than the validation and test datasets.

**Figure 7:**
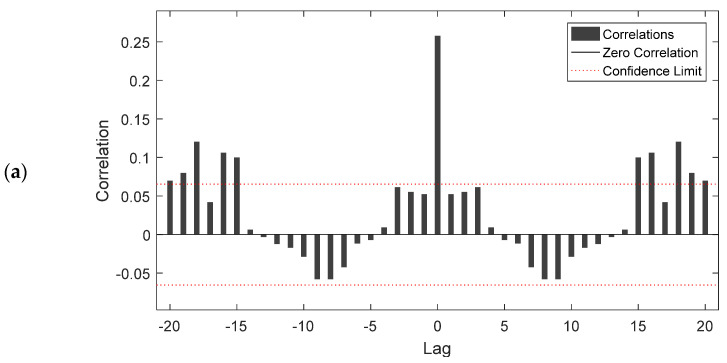

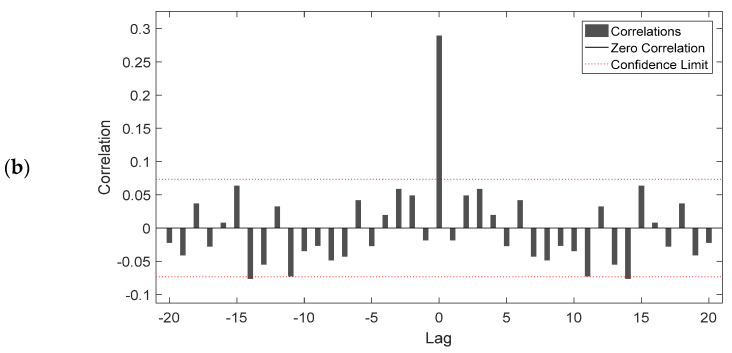
Autocorrelation values of the NARX (**a**) and the FTD (**b**) model in respect to different time lags and related confidence limits.

Figure 7 shows the autocorrelation function of error 1 for the NARX (Figure 7a) and the FTD (Figure 7b) model, respectively, in relation to different time lags and related confidence limits. At zero lag, the autocorrelation equlled the mse, while for the succeeding lagged autocorrelations, the correlation coefficient did not exceed the upper and lower confidence intervals, except for some cases. This means that most of the lagged self-correlated values, for both models, were small and in acceptable levels, considering that values that lagged from zero until 15 (weeks) were between the upper and the lower confidence intervals.

### 3.4. Soft Computing Algorithm and Extension for Decision Support

**Figure 8:**
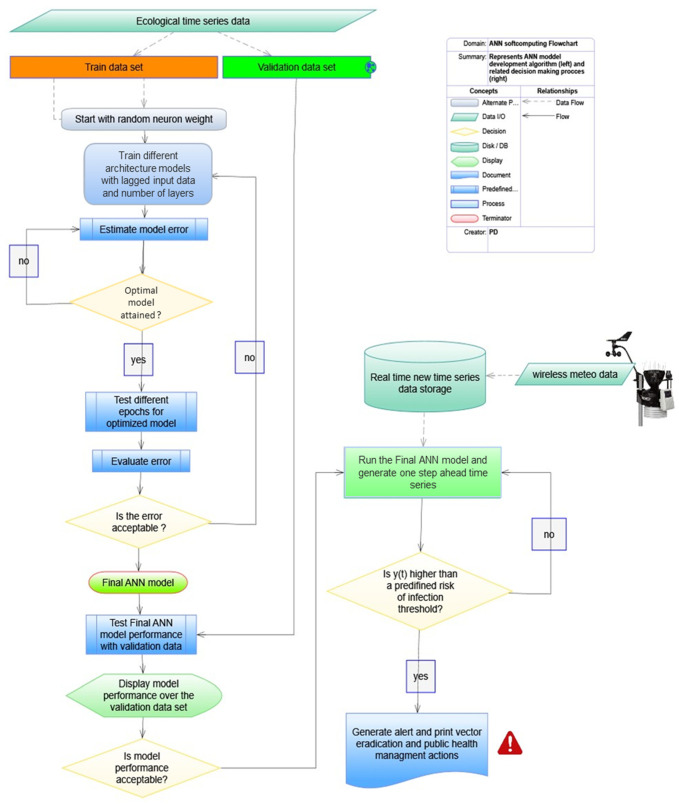
Graphical illustration of the logical operations followed to develop the dynamics autoregressive ANNs models for predicting adult mosquito population dynamics (**left**). Real-time data can be used later to forecast the arthropod vector population dynamics based on the calibrated ANN model that has been developed or to be retrained under new circumstances (**right**).

Figure 8 shows the procedure that was followed to develop the ANNs model, as well as an extension which can be potentially be generated to be used for vector eradication programs and related health-management actions decision making. The ANNs model development provides a robust method for analyzing the past data and to later be used to forecast the arthropod vector population dynamics in respect to real-time data (i.e., temperatures).

The algorithm describes the steps, initial choices, and related routines (i.e., loops-decisions) that were used to end up with the final feedforward ANN model with a tapped delay line at the input (i.e., one time step: one week).

First, data preparation and preliminary testing was performed to decide upon the best data set to be used for model training and validation. The validation datasets consisted of the sample of data held back from training, while the test data set was used for fine tuning (optimizing) the ANN model hyper-parameters (i.e., taking weights of the trained ANN and using it as initialization for a new model being trained, etc.).

Initially, the process started by selecting a small number of neurons (i.e., 5–10) in respect to some initial random weights (e.g., supervised learning) for the synapses, and each time the network was trained, it resulted in a different solution due to the different initial weight and bias values, as well as network properties (e.g., number of neurons). Note that different divisions of data into training, validation, and testing may also have resulted in different model performance. The model was retrained several times to ensure it had good accuracy towards an optimal solution based on an error measurement. The error, as shown in the material section, was defined as the difference of the output of the ANN and the pre-specified external desired data series. The error was estimated for different ANN structures related to the number of hidden layers to derive the final model which performed best. The optimized final model can be fed with new data *per se*, or the model could be retrained to predict the values of future time steps.

## 4. Discussion

ANNs have been used to model complex and abrupt time-varying data and are known to provide completive results to traditional time series models [60], although they have been rarely used in entomology [44]. ANNs have the potential to predict fluctuations in mosquito numbers (especially the extreme values) better than traditional statistical techniques [61]. Moreover, recurrent neural networks (RNNs), in particular, are at the forefront of the research community’s efforts, as they can replace traditional multivariate linear regression models with non-linear models [53].

In this study, we applied two different recurrent ANNs models to describe the adult population dynamics of *Culex sp.*, which helps to describe the population dynamics of this medically important mosquito. In both models, temperature, which is the most detrimental environmental factor, was selected as the input variable to both models. The NARX recurrent network received the sequence of two external inputs as well as the recurrent output layer state, while the FTD network consisted of a feed forward network with a tapped delay line at the input.

To the best of our knowledge, the development and application of the current ANNs is one of the first of its kind in modeling arthropod vector dynamics, although a practical limitation of the current work is that we used a limited dataset to train, validate, and test the model. However, this was expected when we designed the current study, considering that in temperate climates, insect populations are regularly observed weekly over a short period of time of mosquito activity. Thus, the particular dataset should be considered as representative in developing this sort of arthropod population prediction model.

Considering the soft computing approach, both networks belong to the general class of recurrent dynamic neural networks (RNNs), which, in contrast to ANNs, are designed to take a series of inputs with no predetermined limit on size and to memorize prior inputs while generating an output. Although the NARX model predicted slightly better in compared to the FTD model, the differences in model performance were low in general. A practical implication of this fact in model development is that temperature can be used as the main input contributor of the ANN to predict population abundance and that the inclusion of previous mosquito population abundance does not dramatically improve the model performance. As a result, the advantage of the FTD over the NARX neural network model is that it can be applied in cases where past values of mosquito abundance are not available.

From a practical standpoint, the purpose of both models was to predict the next value of mosquito abundance, taking into account past values of input variables as well all past predictions of the model to improve its forecasting efficacy. Based on the results, of both models, the mosquito populations had a certain period of high activity in a temperate climate (i.e., population peaks of high abundance), which can be further used to initiate specific management actions against periods of high activity of mosquito adults.

Moreover, recent studies applying univariate ANNs to model underlying population abundance trajectories do not take in to account the effect of other dynamics variables to model population processes with structure or interactions [45,62]. For cold-blooded species, however, such as mosquitoes and other arthropod vectors, temperature is considered as a predominant factor affecting their life history traits [49,63,64]. This was the main reason why, in this study, we considered only temperature, particularly, as the major exogenous driving factor to model the mosquito seasonal population dynamics.

Meta-analytic results, for instance, performed on another mosquito species, *Aedes aegypti*, indicated that the environmental factor of temperature was sufficient to explain development rate variability and that factors such as diet should never be considered with the exclusion of temperature in modeling development [65]. A preliminary study actually indicated a very low correlation between mosquito abundance and other climatic variables, such as rain, relative humidity, and wind speed [20]. In addition, related dengue virus transmission is influenced by the amplitude and pattern of daily temperature variation [66], while development and survival rate of both *Anopheles* mosquitoes and the *Plasmodium* parasites that cause malaria depend on temperature, making this a potential driver of mosquito population dynamics and malaria transmission [67]. In addition, for the West Nile Virus in *Culex pipiens*, increasing temperatures may accelerate transmission of WNV, as demonstrated by Kilpatrick et al. (2008) [68].

However, due to the vague nature of the ANN, it is rather difficult to answer the question of how temperature and *Culex* dynamics interact as in the case of traditional insect population models. To date, although neural networks have relative analogies to regression models, in which coefficients of interaction parameters are replaced by weights, conceptually, they differ. To put forward an ANN is closer to an additive model, which uses non-parametric regression methods and alternates conditional expectations algorithms for an optimal, smother transformation between response and prediction variables. However, in the case of the ANN, a single layer learns how combinations of the input variables are related to the output variables (i.e., a type of first-order interaction but non-linear). Moreover, the autoregressive ANN models, implied in the current study, use, additionally to input variables, the model outputs in a feedback loop to improve its accuracy as in the case of autoregressive (AR) models.

From an ecological standpoint, the detection of first-order feedbacks, through autocorrelation functions, is considered as the result of interspecies interaction and is an indication to be considered in modelling population dynamics. Nevertheless, the construction of well-ordered models is not only important in discovering the forces that drive vector population dynamics, but also a prerequisite for the initiation of management actions from the point of view of public health.

The understanding of mosquito phenology and the description of its population dynamics is essential for the prevention of vector-borne diseases and to initiate proper management actions [69,70,71]. Thus, to facilitate this understanding, it seems reasonable to build mathematical models of increasing complexity that reflect some true state of the time evolution and dynamics of natural populations of mosquitoes [21]. In specific, artificial neuron networks (ANNs) may contribute very significantly to improvement of the accuracy of population data description, particularly that of mosquito abundances, which, due to their specific life cycle, are most often characterized by abrupt outbreaks.

To date, ANNs were originally proposed as a mathematical model of complex simulation of the functioning of the human brain [72,73]. The brain structure is such that it enables data to be processed in parallel and lifelong learning through interaction with environment. ANNs create artificial intelligence (AI) and mimic this biological property by putting forward input signals, stimulating the network’s capability to learn and recognize patterns. In contrast to traditional statistical models (i.e., autoregressive models, multivariate regression models) that have been used to model arthropod vector abundance and related disease dynamics [74,75,76,77,78], the ANNs models that have been applied in the current study have, as a main asset, the ability of their neurons (i.e., sub-models of different weights) working simultaneously, but independently from each other [79]. Particularly in the cases where the outcome variable (i.e., here, the mosquito abundance) is affected by more factors (i.e., temperature, previous mosquito abundance, and more), these can be independently introduced and taken into account by the network in terms of its weight during the learning process (or training).

One other advantage of using ANNs models over time series models (linear and non-linear) is the fact that the cases where predictions are performed form a random sample from the same population as the time periods about which one makes the prediction. Additionally, the performance of autoregressive time series models is also affected in situations of limited data availability where the true shape of data distribution is unknown [80,81,82].

On the other hand, one limitation of ANN models is that there is no set method for the construction of the network architecture [28]. For instance, the development of the final model structure, which was presented in the current study, was the result of numerous prior combinations of candidate ANNs model structures (i.e., number of neurons and hidden layers) and input variables (i.e., lagged climate and mosquito abundance data). One other explanatory limitation of ANNs is that the analysis generates weights, instead of standardized coefficient parameters, which are difficult to interpret and often not as present as they are in regression analysis [83].

Therefore, one of the most criticized features in ANNs is the lack of interpretability at the level of individual variables [84]. Moreover, since the ANN learning performance was checked against the disjoint set of data that was available (i.e., test set), it is of fundamental importance to choose an appropriate training set size and to provide representative coverage of all possible conditions for modeling.

From an ecological perspective, in this work, we considered only the dynamics of adults and not that of the immature stages, which may also be affected by temperature [85]. However, the available data of immature stages are hard to systematically collect, and, probably, therefore, related analyses which explicitly incorporate other growth stages in time series population models are rather absent from literature. Nevertheless, by implication, the role of ANNs models is to provide a functional framework of an empirical relationship between system inputs and outputs (i.e., black box, Olden and Jackson, 2002) [86], rather than to abstract a strict deterministic description of the process itself. Therefore, the aim of the current study was to introduce a novel modeling approach to capture the non-linear nature of mosquito population dynamics in relation to temperature, rather than to describe how specific environmental factors at one life stage affect that stage’s performance and subsequent life stages (i.e., carry-over effects).

Nevertheless, compared to existing mathematical models used to describe ecological time-series population dynamics, the proposed ANNs provide a robust non-linear modelling framework without the need for any prior knowledge and assumption between the input and output variables [87,88]. Additionally, compared to other time-series models, a notable advantage of neural network modelling is its better prediction accuracy when the available time series are noisy and short, and in relation to the process, there is a lack of understanding of the process underlying the population fluctuations [88]. To date, although non-linear time series models have made notable progress, such as conditionally heteroscedastic models, threshold autoregressive models do not necessarily improve model predictions over older models [81,82,87]. Thus, the proposed ANNs models may provide a practical data-driven alternative ecological time series modeling approach, without providing restricted causal rules and related parameter estimates in cases where the underlying population process is not fully understood.

Moreover, development rates of mosquitoes are known to vary with respect to many abiotic and biotic factors, including temperature, resource availability, and intraspecific competition. However, meta-analytic results performed on another mosquito species, *Aedes aegypti*, indicated that the environmental factor of temperature was sufficient to explain development rate variability and that factors such as diet should never be considered with the exclusion of temperature in modeling development [65]. In addition, related dengue virus transmission is influenced by the amplitude and pattern of daily temperature variation [66]. Nevertheless, despite temperature being the most influential factor to include in a predictive model of mosquitoes, other climatic variables, such as wind, which are limiting for mosquito flying and their feeding behavior, have not been included in the current work. Therefore, we are looking forward to study whether more climatic variables are correlated with mosquito populations and if the model performances are improved by the inclusion of additional ecological time series. Moreover, the proposed methods could be extended and the models retrained to evaluate the predictions of other arthropod/disease vector population dynamics. Furthermore, despite the above limitations, the strength of the ANNs used in this study was the absence of normality assumptions and their ability to find and describe, with acceptable precision, the dynamics of the mosquito population patterns despite limited data. Therefore, although many models have been developed to examine vector population dynamics, the proposed ANNs modeling approach has many potentials to be further improved and used to predict mosquito vector dynamics for decision support [89]. Furthermore, more exploration is required into the prediction of vector-borne disease dynamics incorporating more variables to improve the accuracy in real practice.

## 5. Conclusions

In this study, the use of ANNs in modeling *Culex sp*. population dynamics was preferred over other techniques, since it is better employed to perform population predictions that handle short and, in some cases, incomplete data. In particular, for short time changes, as in the case of modeling mosquito abundance in temperate climates, the non-linear and semi-parametric nature of artificial neural networks (ANNs) are very promising for performing multivariate dynamic forecasts. The major advantage of the FTD over the NARX neural network was that it did not require dynamic backpropagation to compute the network gradient, because the tapped delay line appeared only at the input of the network and did not contain feedback loops. On the other hand, the NARX neural network provided an attractive method for resolving the particular empirical *Culex sp.* population prediction problem through real sensing of lagged mosquito abundances and temperature. Although much more work needs to be conducted to assess the effect of meaningful time delays and environmental variables on other developmental stages, the current modeling approach provides a general basis to be used in modeling mosquito surveillance data to predict arthropod vector dynamics. Additionally, this study has a key role to play in designing sustainable control and public health elimination strategies of medically important arthropod vectors and the development of public health decision support systems.

## Data Availability

Publically available datasets were analyzed in this study. This data can be found here: [http://data.europa.eu (accessed on 3 May 2019)] and here: [http://stratus.meteo.noa.gr/front (accessed on 2 April 2020)].
